# New *PCNT* candidate missense variant in a patient with oral and maxillofacial osteodysplasia: a case report

**DOI:** 10.1186/s12881-019-0858-z

**Published:** 2019-07-16

**Authors:** Ken-ichi Aoyama, Minoru Kimura, Hiroshi Yamazaki, Masahiro Uchibori, Rena Kojima, Yuko Osawa, Kazuyoshi Hosomichi, Yoshihide Ota, Masayuki Tanaka, Shiro Yamada, Gen Nishimura

**Affiliations:** 1 Department of Oral and Maxillofacial Surgery, Tokai University School of Medicine, 143 Shimokasuya, Isehara, Kanagawa 259-1193 Japan; 2 Department of Molecular Life Science, Tokai University School of Medicine, 143 Shimokasuya, Isehara, Kanagawa 259-1193 Japan; 3 Department of Oral and Maxillofacial Surgery, Tokai University Oiso Hospital, 21-1 Gakkyo, Oiso, Kanagawa 259-0114 Japan; 4 Department of Bioinformatics and Genomics, Kanazawa University, 13-1 Takara-machi, Kanazawa, Ishikawa 920-8640 Japan; 5 Department of Bioinformatics, Support Center for Medical Research and Education, Tokai University School of Medicine, 143 Shimokasuya, Isehara, Kanagawa 259-1193 Japan; 6 Department of Pediatrics, Tokai University Oiso Hospital, 21-1 Gakkyo, Oiso, Kanagawa 259-0114 Japan; 7 Department of Pediatric Imaging, Tokyo Metropolitan Children’s Medical Center, 2-8-29 Musashidai, Fuchu, Tokyo 183-8561 Japan

**Keywords:** Local osteodysplasia, Oral and maxillofacial bones, PCNT, Pericentrin, Whole exome sequencing

## Abstract

**Background:**

Osteodysplasia of the oral and maxillofacial bone is generally accompanied by systemic bone abnormalities (such as short stature, joint contracture) or other systemic abnormalities (such as renal, dermatological, cardiovascular, optic, or hearing disorders). However, it does not always present this way. Recent reports have suggested that genome-wide sequencing is an effective method for identifying rare or new disorders. Here, we performed whole-exome sequencing (WES) in a patient with a unique form of acquired, local osteodysplasia of the oral and maxillofacial region.

**Case presentation:**

A 46-year-old woman presented to our hospital with the complaint of gradually moving mandibular teeth (for 6 months), changing facial appearance, and acquired osteolysis of the oral and maxillofacial bones, showing mandibular hypoplasia without family history. Upon skeletal examination, there were no abnormal findings outside of the oral and maxillofacial area; the patient had a height of 157 cm and bone mineral density (according to dual energy x-ray absorptiometry) of 90%. Results of blood and urine tests, including evaluation of bone metabolism markers and neurological and cardiovascular examinations, were normal. We performed WES of genomic DNA extracted from the blood of this patient and her mother, who did not have the disease, as a negative control. We identified 83 new missense variants in the patient, not detected in her mother, including a candidate single nucleotide variant in exon 14 of *PCNT* (pericentrin). Critical homozygous or compound heterozygous variants in *PCNT* are a known cause of microcephalic osteodysplastic primordial dwarfism type II accompanied by mandibular hypoplasia, which is similar to the maxillofacial phenotype in this patient.

**Conclusions:**

Protein simulations performed using Polymorphism Phenotyping v2 and Combined Annotation Dependent Depletion software indicated that this missense variant is likely to disrupt the PCNT protein structure. These results suggest that this is a new form of osteolysis related to this *PCNT* variant.

## Background

Osteodysplasia of the oral and maxillofacial bone is generally accompanied by systemic metabolic bone disease or other systemic abnormalities, such as cardiac malformations or neurological disorders [1–3]. Acquired local osteodysplasia without metabolic disease is usually accompanied by the presence of abnormal soft tissues, including malignant tumors [3]. However, acquired local osteodysplasia is rarely reported.

The relative affordability and accessibility of genome-wide sequencing have facilitated the development of family-based genomic analysis, resulting in an explosion of gene discovery and diagnosis of rare diseases [4]. However, for many congenital malformations, identification of the causative mutation by whole-genome sequencing or whole-exome sequencing (WES) has been challenging [5]. Nonetheless, recent studies have identified specific gene variants in patients with congenitally acquired skeletal disorders, and genome-wide sequencing is a potent technique for the identification of variants implicated in unknown disorders [6].

Here, we performed WES in a patient with a potentially unknown skeletal disorder involving oral and maxillofacial acquired local osteodysplasia without metabolic disease or soft tissue around the bone lesions.

## Case presentation

### Case history

In 2015, a 46-year-old woman presented to the Department of Oral and Maxillofacial Surgery of Tokai University Oiso Hospital, Kanagawa, Japan, with the complaint of gradually moving mandibular teeth (for 6 months) and changing facial appearance. She had been referred by her family dentist, who had noticed the early stages of mandibular bone loss 9 years prior. She had received no treatment other than periodontal therapy performed by her dentist.

### Family history

The patient was the youngest of two children. Her mother and father were 32 and 36 years old, respectively, at the time of her birth. Her father died in an accident at the age of 40. Her sister did not exhibit similar symptoms or present with facial malformation.

### Medical history

Birth weight and intelligence level were normal. The patient’s history was remarkable for 10 episodes of bacterial meningitis, which occurred between 31 and 39 years of age. She was premenopausal at presentation.

### Physical findings

We consulted an orthopedic surgeon and genetic physician with the intent of performing a comprehensive screening for systemic disorders. Upon skeletal examination, there were no abnormal findings outside of the oral and maxillofacial area; the patient had a height of 157 cm and bone mineral density (according to dual energy x-ray absorptiometry) of 90%. Results of blood and urine tests for bone metabolism markers, including calcium (9.8 mg/dL), phosphorous (4.4 mg/dL), alkaline phosphatase (284 IU), 25-OH-Vitamin D (55 pg/mL), and collagen cross-linked N-telopeptide (18.4 nmol), as well as neurological and cardiovascular examinations, were normal.

**Fig. 1:**
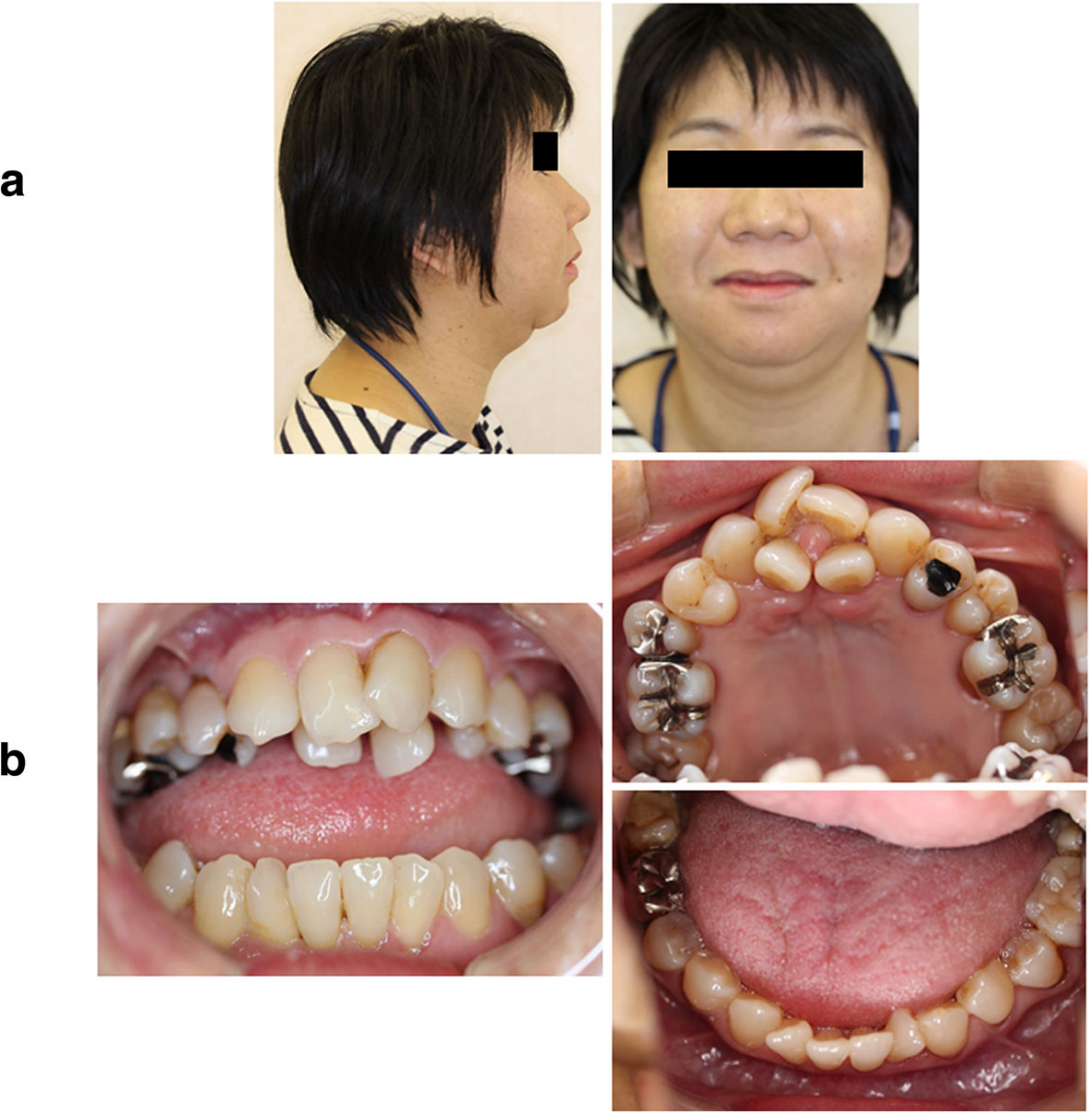
Patient imaging data. **a** Photographs from extraoral examination reveal mandibular hypoplasia but no asymmetry. **b** Photographs from intraoral examination reveal an anterior open bite (inter-incisor distance: 23 mm) and early contact of the second molars. The upper teeth are crowded without a cleft palate; the lower teeth are not crowded

Extraoral examination revealed mandibular hypoplasia but no asymmetry (Fig. 1a). Intraoral examination revealed an anterior open bite (inter-incisor distance: 23 mm) and no crowding of the mandibular teeth. There were no abnormal findings pertaining to the oral mucosa (Fig. 1b).

### Radiographic findings

**Fig. 2:**
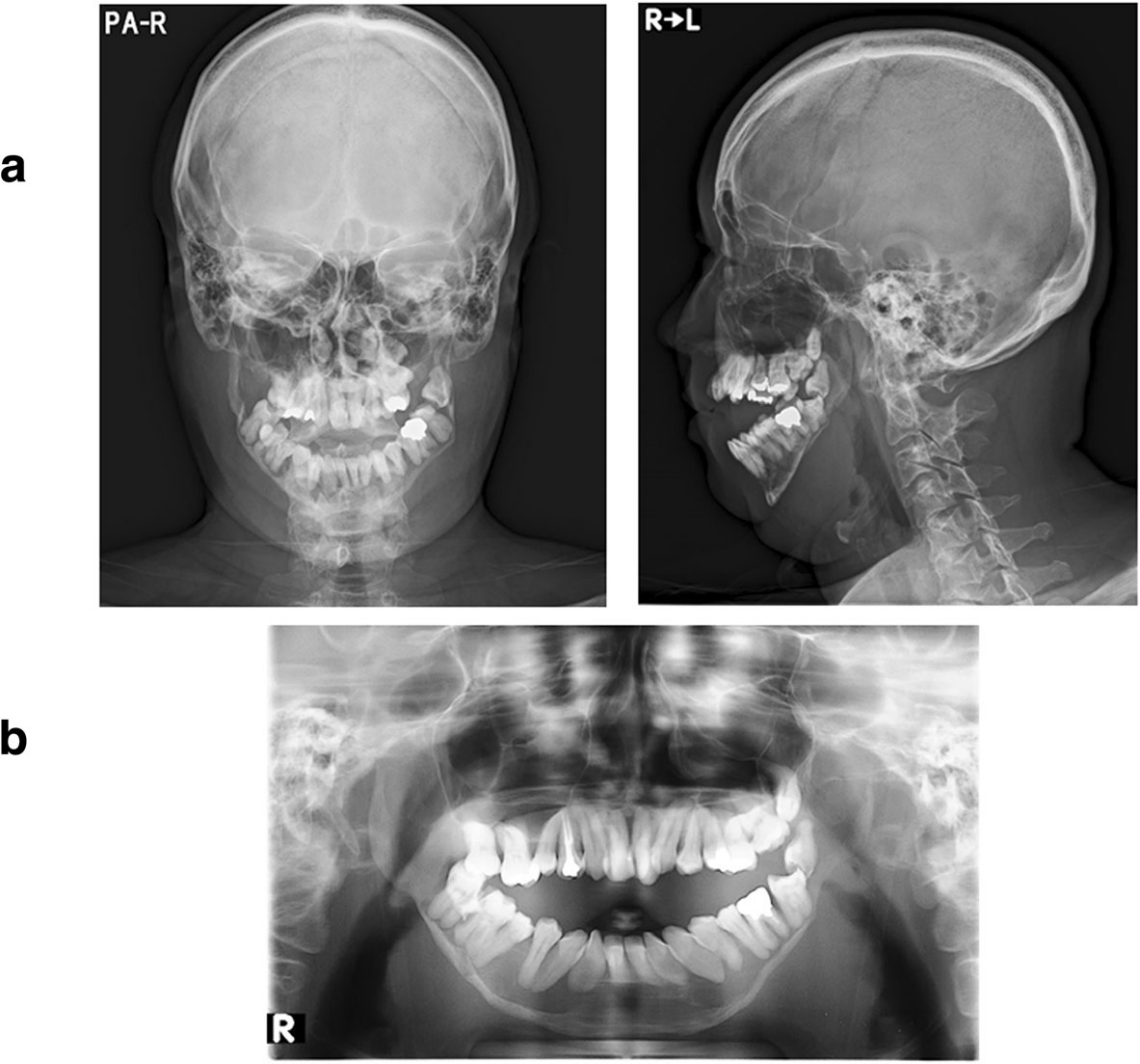
X-ray images. **a** Posteroanterior and lateral skull views do not reveal morphological abnormalities of the head or face height and width. **b** A panoramic radiographic image shows axial and coronal thinning of the alveolar bone in the maxilla and the entire mandibular (condyle, angle, body, and alveolar) bone

**Fig. 3:**
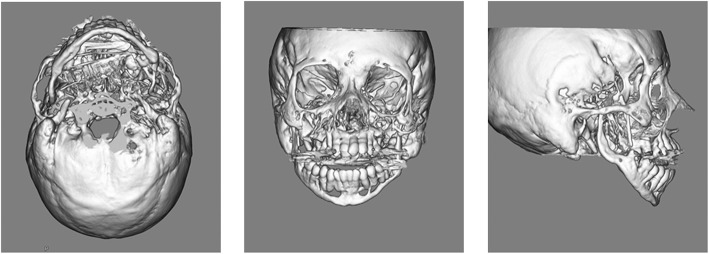
Computed tomography imaging. Imaging shows axial and coronal thinness of the alveolar bone, anterior wall of the maxillary sinus in the maxilla, and the entire mandibular (condyle, angle, body, and alveolar) bone

**Fig. 4:**
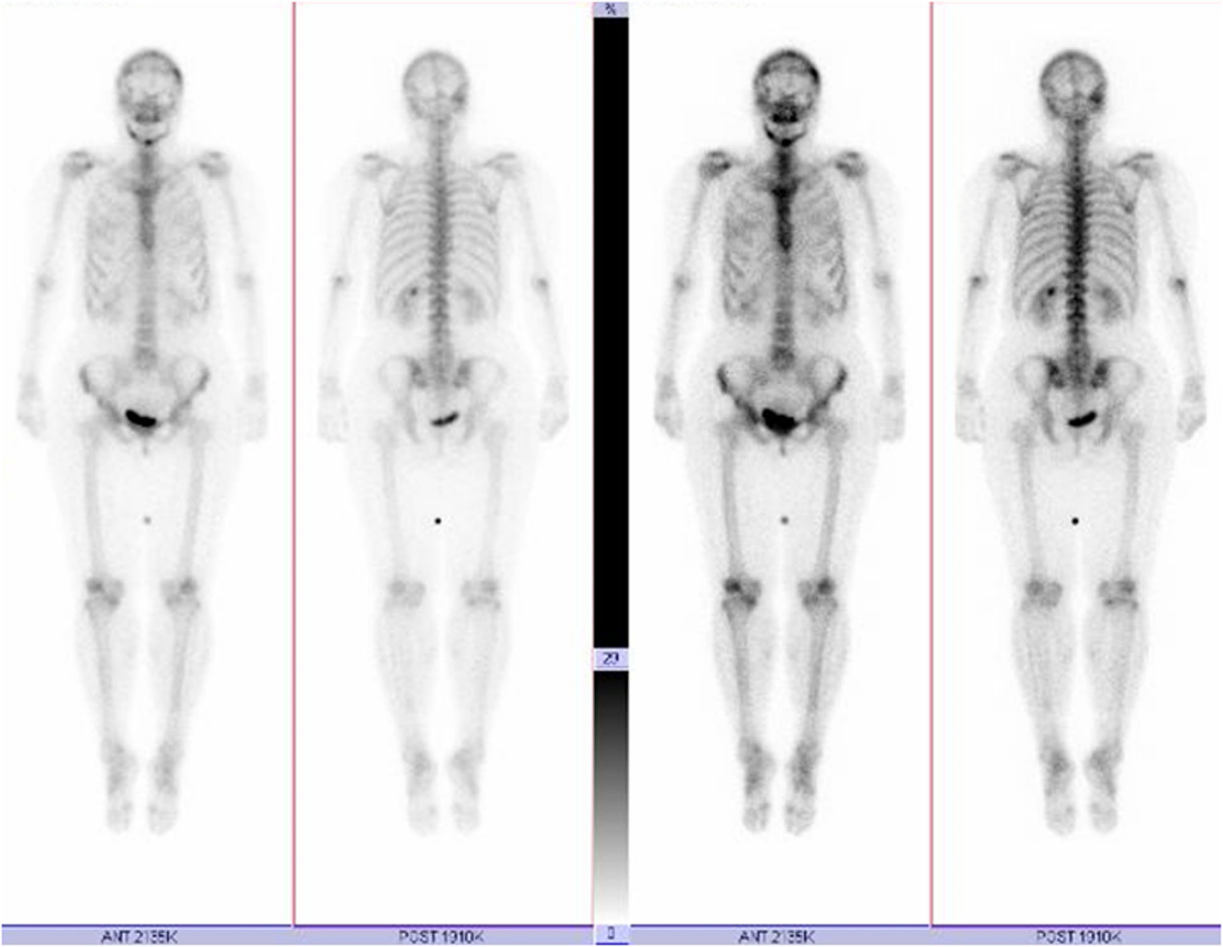
99mTc bone scintigraphy shows tracer uptake in the maxillary and mandibular bones

Posteroanterior and lateral radiographs of the skull did not reveal any abnormal morphology of the head or facial height and width (Fig. 2a). Panoramic radiographic images and computed tomography scans showed axial and coronal thinning of the alveolar bone, anterior wall of the maxillary sinus in the maxilla, and the entire mandibular (condyle, angle, body, and alveolar) bone (Figs. 2b and 3). Magnetic resonance imaging did not show any soft tissue masses in the maxillofacial area (data not shown). Technetium (99mTc) bone scintigraphy showed tracer uptake in the maxillary and mandibular bones (Fig. 4).

### DNA extraction, library preparation, and sequencing

Genomic DNA was extracted from 100 μL of whole blood from the patient and her mother (as a negative control) using the DNeasy Blood & Tissue Kit (Qiagen, Hilden, Germany) following the manufacturer’s recommendations. Initial DNA sample quality assessment, library preparation, and sequencing were conducted by GENEWIZ, Inc. (South Plainfield, NJ, USA). The SureSelectXT Target Enrichment System for Illumina Paired-End Multiplexed Sequencing Library and SureSelect Human All Exon V6 bait library (Agilent, Santa Clara, CA, USA) were used for target enrichment and DNA library preparation following the manufacturer’s recommendations.

The sequencing library was loaded onto an Illumina HiSeq instrument (San Diego, CA, USA) according to the manufacturer’s instructions. Raw sequence data (.bcl files) generated from the Illumina HiSeq instrument were converted into fastq files and de-multiplexed using bcl2fastq software 2.17 (Illumina). One mismatch was allowed for index sequence identification. The data of whole exome sequence was assigned on The DNA Data Bank of Japan Center (NBDC number: hum0190) [7].

The average coverage depth of the entire panel was 137×, and 99.9% of targeted bases were covered by sequence reads at a depth of at least 20 × .

### Candidate gene approach and gene annotation

Sequence reads were aligned using BWA (version 0.7.12) [doi:10.1093/bioinformatics/btp324]. Local realignment and base quality recalibration was performed using GATK (version 3.5) [doi: 10.1101/g.107524.110]. Variant calling was performed with SAMtools (version 1.3.1) [doi.org/10.1093/bioinformatics/btp352] [doi.org/10.1093/bioinformatics/btr509]. Variants were annotated using the ANNOVAR tool [doi: 10.1093/nar/gkq603].

Only non-synonymous sequence differences between the mother and patient in the sequenced genes were analyzed. Candidate gene sequencing revealed 83 heterozygous variants in 79 genes, which were confirmed by Sanger sequencing (Table 1). These single-nucleotide variants (SNVs) were not present in any of the queried population databases [(Integrative Japanese Genome Variation Databas (iJGVD), The International Genome Sample Resource and Providing ongoing support for the 1000 Genomes Project data (1000 Genomes), The Exome Aggregation Consortium (ExAC), snp138, Human Genetic Variation Database (HGVD), and The Genome Aggregation Database (gnomAD)]. Among the 83 heterozygous variants, 12 SNVs in 11 genes (*AHNAK, CCDC15, COBL, DCAF5, MCF2L, NSRP1, NSRP1, PCNT, RMDN3,* and *TTYH2*) were considered to be “probably damaging” according to Polymorphism Phenotyping v2 (PolyPhen-2) (http://genetics.bwh.harvard.edu/pph2/) and Combined Annotation Dependent Depletion (CADD) [https://cadd.gs.washington.edu/]. These databases predict the potential effect of an amino acid substitution on the structure and function of a human protein using straightforward physical and comparative considerations [8].

**Table 1:** Detected nonsynonymous variants in the patient revealed by candidate gene sequencing and confirmed by Sanger sequencing

Chromosome	Start	End	Refseq	Altered sequence	Gene symbol	Exonic mutation type
chr2	69746096	69746096	T	C	AAK1	nonsynonymous SNV
chr1	229694116	229694116	C	G	ABCB10	nonsynonymous SNV
chr7	150728344	150728344	G	T	ABCB8	nonsynonymous SNV
chr7	45614282	45614282	C	G	ADCY1	nonsynonymous SNV
chr11	62298351	62298351	C	G	AHNAK	nonsynonymous SNV
chr5	74921513	74921513	C	A	ANKDD1B	nonsynonymous SNV
chr5	10649897	10649897	G	A	ANKRD33B	nonsynonymous SNV
chr5	112175876	112175876	C	T	APC	stopgain
chr5	112175918	112175918	A	G	APC	nonsynonymous SNV
chr5	148980794	148980794	G	T	ARHGEF37	nonsynonymous SNV
chr3	130569767	130569767	G	A	ATP2C1	nonsynonymous SNV
chr1	1535392	1535392	G	A	C1orf233	nonsynonymous SNV
chr11	124829898	124829898	C	A	CCDC15	nonsynonymous SNV
chr11	46784694	46784694	G	T	CKAP5	stopgain
chr4	141315195	141315195	C	A	CLGN	stopgain
chr7	51092806	51092806	C	A	COBL	nonsynonymous SNV
chr4	15005450	15005450	G	A	CPEB2	nonsynonymous SNV
chr20	56075374	56075374	G	T	CTCFL	nonsynonymous SNV
chr12	58220819	58220819	A	G	CTDSP2	nonsynonymous SNV
chr12	58220827	58220827	G	C	CTDSP2	nonsynonymous SNV
chr14	59104973	59104973	C	A	DACT1	nonsynonymous SNV
chr14	69589062	69589062	C	A	DCAF5	nonsynonymous SNV
chr4	88536277	88536277	C	A	DSPP	nonsynonymous SNV
chr17	37101383	37101383	G	T	FBXO47	nonsynonymous SNV
chr12	32791811	32791811	A	G	FGD4	nonsynonymous SNV
chr4	123748237	123748237	G	T	FGF2	stopgain
chr6	41565667	41565667	G	T	FOXP4	nonsynonymous SNV
chr6	146678724	146678724	C	T	GRM1	nonsynonymous SNV
chr9	135553823	135553823	A	C	GTF3C4	nonsynonymous SNV
chrX	80370472	80370472	A	C	HMGN5	nonsynonymous SNV
chr20	43034704	43034704	C	A	HNF4A	nonsynonymous SNV
chr7	141401904	141401904	G	T	KIAA1147	nonsynonymous SNV
chr1	66091850	66091850	T	–	LEPR	frameshift deletion
chr4	83905358	83905358	T	C	LIN54	nonsynonymous SNV
chr2	100938297	100938297	G	A	LONRF2	nonsynonymous SNV
chr5	121406215	121406215	C	A	LOX	nonsynonymous SNV
chr15	101606383	101606383	C	A	LRRK1	nonsynonymous SNV
chr13	113718710	113718710	C	G	MCF2L	nonsynonymous SNV
chr11	86161390	86161390	T	C	ME3	nonsynonymous SNV
chr5	79961093	79961093	C	A	MSH3	nonsynonymous SNV
chr3	130947458	130947458	G	–	NEK11	frameshift deletion
chr3	52797604	52797604	C	T	NEK4	nonsynonymous SNV
chrX	17394002	17394002	C	T	NHS	nonsynonymous SNV
chr17	28506267	28506267	G	A	NSRP1	nonsynonymous SNV
chr17	28507941	28507941	C	A	NSRP1	nonsynonymous SNV
chr11	6913128	6913128	T	A	OR2D2	nonsynonymous SNV
chr6	163733852	163733852	A	T	PACRG	nonsynonymous SNV
chr6	163733870	163733870	G	C	PACRG	nonsynonymous SNV
chr21	47783755	47783755	T	C	PCNT	nonsynonymous SNV
chr12	118574117	118574117	G	T	PEBP1	nonsynonymous SNV
chr1	64059254	64059254	G	T	PGM1	nonsynonymous SNV
chr3	111688538	111688538	C	A	PHLDB2	nonsynonymous SNV
chr14	53184835	53184835	G	T	PSMC6	nonsynonymous SNV
chr20	49196452	49196452	C	–	PTPN1	stopgain
chr2	20497410	20497410	C	A	PUM2	nonsynonymous SNV
chr19	913148	913148	G	T	R3HDM4	nonsynonymous SNV
chr17	80009540	80009540	G	T	RFNG	nonsynonymous SNV
chr15	41043685	41043685	T	A	RMDN3	nonsynonymous SNV
chr19	47673139	47673139	C	T	SAE1	nonsynonymous SNV
chr19	50154308	50154308	C	A	SCAF1	nonsynonymous SNV
chr3	38674533	38674533	G	A	SCN5A	nonsynonymous SNV
chr7	94227307	94227307	G	T	SGCE	nonsynonymous SNV
chr21	38120265	38120265	C	A	SIM2	nonsynonymous SNV
chr6	3456742	3456742	G	A	SLC22A23	nonsynonymous SNV
chr1	158590126	158590126	T	A	SPTA1	nonsynonymous SNV
chr2	45812904	45812904	C	A	SRBD1	stopgain
chr13	75900532	75900532	C	T	TBC1D4	nonsynonymous SNV
chr14	104431776	104431776	G	A	TDRD9	nonsynonymous SNV
chr8	23003284	23003284	G	C	TNFRSF10D	nonsynonymous SNV
chr8	23003285	23003285	T	A	TNFRSF10D	nonsynonymous SNV
chr3	189590767	189590767	G	T	TP63	nonsynonymous SNV
chr3	39152470	39152470	A	G	TTC21A	nonsynonymous SNV
chr2	179417341	179417341	G	T	TTN	nonsynonymous SNV
chr2	179590749	179590750	GG	–	TTN	frameshift deletion
chr17	72246413	72246413	C	T	TTYH2	nonsynonymous SNV
chr16	84806169	84806170	CT	–	USP10	frameshift deletion
chr3	49349901	49349901	C	T	USP4	nonsynonymous SNV
chr5	76373354	76373354	G	T	ZBED3	nonsynonymous SNV
chr2	187364925	187364925	C	A	ZC3H15	nonsynonymous SNV
chr9	109688202	109688202	A	G	ZNF462	nonsynonymous SNV
chr9	99537070	99537070	C	A	ZNF510	nonsynonymous SNV
chr5	60628634	60628634	G	A	ZSWIM6	nonsynonymous SNV

**Fig. 5:**
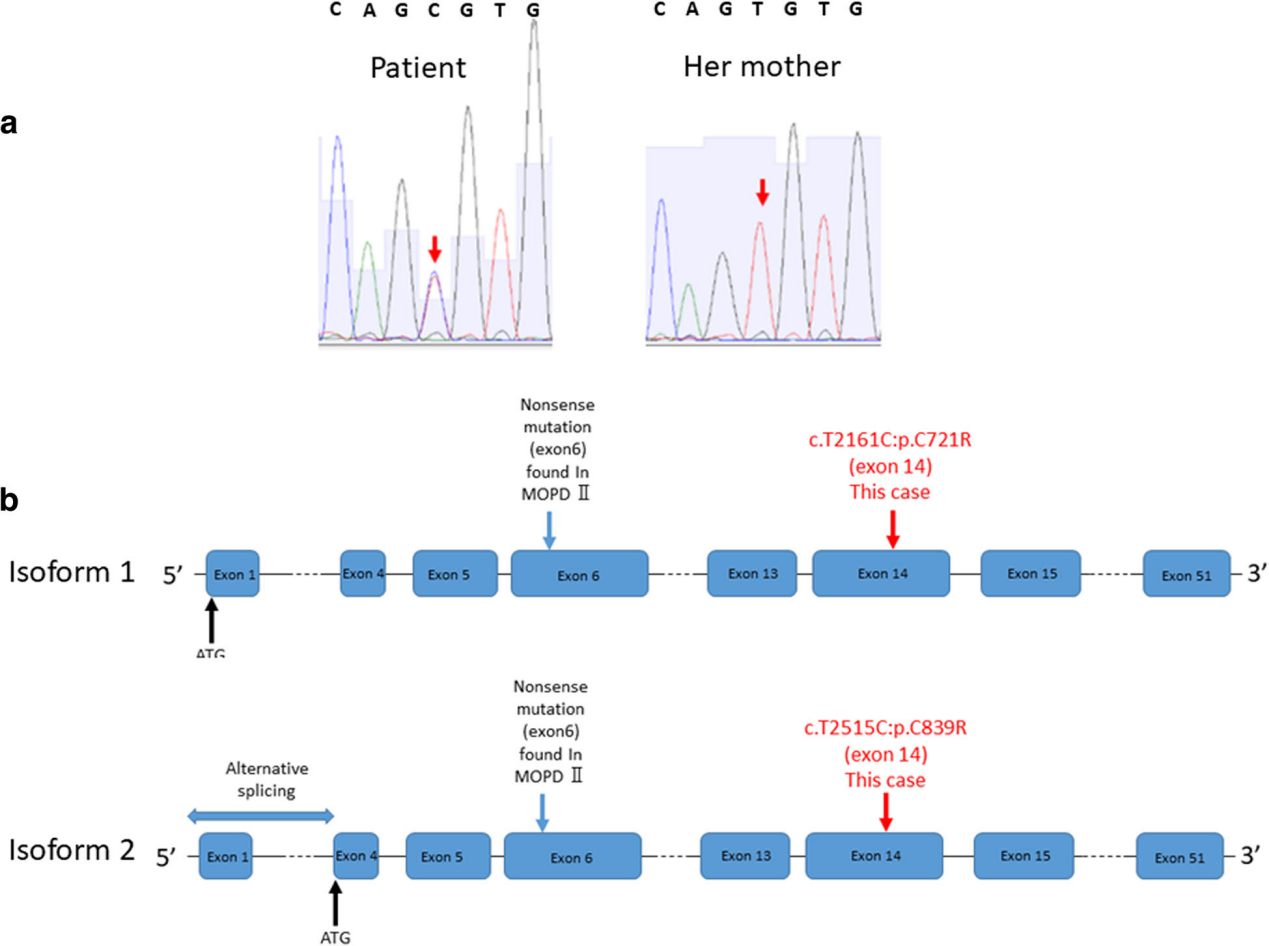
Missense variant present in exon 14. The variant was determined to be *PCNT*: NM_006031, exon 14, c.T2515C, p.C839R; NM_001315529, exon 14, c.T2161C, p.C721R. **a** Sanger sequencing results for the patient and her mother. **b** Mutation map of two isoforms (transcript variants)

The 11 genes with probably damaging SNVs were analyzed using the Genecards*®* human gene database version 4.5 [https://www.genecards.org/]. AHNAK may be involved in diverse processes such as blood-brain barrier formation, cell structure and migration, cardiac calcium channel regulation, and tumor metastasis [9]. COBL may play a role in maintaining intestinal homeostasis [10]. NSRP1 is an mRNA binding protein that has not been associated with any clinical features [11]. The molecular and clinical functions of CCDC15, DCAF5, and RMDN3 have not been clarified according to Genecards*®*. MCF2L is related to the Rho/Rac signaling pathways, which play key roles in cell proliferation, migration, and motility, including in cancer metastasis [12]. Among the 11 genes, only *PCNT* is expressed in the cortex and skeletal muscle. We speculate that the *PCNT* variant (chr21 47783755: NM_006031, exon14, c.2515 T > C, p.839C > R; NM_001315529, exon 14, c.2161 T > C, p.721C > R) in the patient may affect the cell division of osteoblasts or osteoclasts and bone homeostasis in the oral and maxillofacial area. This differs from the result of *PCNT*-null disorders (Fig. 5a). Thus, we considered *PCNT* a candidate gene for this disorder because of its high mRNA expression in the cortex and skeletal muscle and the lack of clinical reports for *AHNAK, CCDC15, COBL, DCAF5, MCF2L, NSRP1, NSRP1, RMDN3*, and *TTYH2*. Notably, p.C721R/p.C839R of PCNT has not yet been reported as a candidate variant for skeletal disorders in the Nosology and Classification of Genetic Skeletal Disorders, which serves as a “master” list of genetic disorders of the skeleton to facilitate diagnosis and to help delineate variants or newly recognized conditions [13].

### Treatment and follow-up

Because this osteolytic disorder had not been previously reported and no specific treatment was indicated, the patient was placed under observation with periodontal maintenance. No progression has been noted in the 2.5 years since diagnosis.

## Discussion and conclusions

Osteolysis in the oral and maxillofacial bones can be a phenotype of various systemic disorders, calcium and phosphorus disorders, hyperparathyroidism, hypoparathyroidism, osteomalacia, osteopenia, osteoporosis, Paget’s disease, and vitamin D deficiency [1, 3, 6, 13, 14]. Congenital osteolysis is often hereditary and accompanied by disturbances in bone metabolism [14]. Major osteolysis can lead to skeletal malformations, such as short stature. Although familial osteodysplasia localized in the mandible was reported in four of six siblings (13 to 23 years old) in 1972, none of the patients had any offspring, and the genetic characteristics of this disease were not investigated [15].

Potential differential diagnoses of osteodysplasia syndromes, which involve bone anomalies in the oral and maxillofacial region, are shown in Table 2 [6, 16]. Gorham’s disease is an acquired condition characterized by local or massive osteolysis that can involve the oral and maxillofacial bones. In this disease, the osteolytic region is often accompanied by soft hemangiomatous or lymphangiomatous tissue [6]. In our patient, although osteolysis was shown to be localized in the oral and maxillofacial region, soft tissue did not appear around the bone lesions. Hence, we were not able to make a diagnosis based on clinical features. Therefore, we performed WES in the patient and her mother, who did not have a bone disorder, and sought to identify gene variants in known skeletal disorder genes and to clarify the genetic basis of this maxillofacial osteolysis.

**Table 2:** Differential diagnoses of osteodysplasia syndromes, which involve bone anomalies in the oral and maxillofacial region

Familial or not	Disruption of genes	Congenital or acquired	Common sites of osteolysis	Features except ostelysis
Sporadic	None	Acquired	Shoulder, Pelvis (not systemic)	Pain, swelling (systemic inframation)
Familial	NOTCH2	Congenital	Metacarpal bones, metatarsal bones, forearm bones (often systemic)	Short stature, optic atrophy, hearing loss
Familial	MAFB	Congenital	Carpals, phalanges of the toes (often systemic)	Renal failure
Sporadic	NPHS2, ACTN4	Congenital	Carpals, phalanges of the toes (not systemic)	Renal failure, hypertension
Familial	MMP2	Congenital	Phalanges, rib, clavicular (not systemic)	Atraumatic painless fracture
Familial	Not identified	Congenital	Phalanges of the fingers (not systemic)	Tabes dorsalis, syringomyelia, spinal cord tumor
Familial	Not identified	Congenital	Phalanges of the fingers and toes (not systemic)	Skin ulcers
Familial	ASAH1	Congenital	Phalanges of the fingers and toes (not systemic)	Painful joint deformity, subcutaneous nodules, hoarseness
Familial	MMP14	Congenital	Phalanges of the fingers and toes (not systemic)	Short stature, severe joint contractures, peripheral corneal opacities

PCNT is one of the calmodulin-binding proteins expressed in the centrosome. PCNT (< 370 kDa) contains a series of coiled-coil domains and localizes specifically to the centrosomes throughout the cell cycle [17]. The protein interacts with the microtubule nucleation component gamma-tubulin and is likely important for normal functioning of the centrosomes, cytoskeleton, and cell-cycle progression. Two transcript variants encoding different isoforms of this gene are annotated in the RefSeq database: NM_001315529.1 and NM_006031.5 [https://www.ncbi.nlm.nih.gov/nuccore/NM_001315529.1 and https://www.ncbi.nlm.nih.gov/nuccore/NM_006031.5]. The domain structure and function around p.C721R/p.C839R have not been determined.

Critical mutations such as homozygous or compound heterozygous mutations in *PCNT* are a known cause of microcephalic osteodysplastic primordial dwarfism type II (MOPD II; Mendelian Inheritance in Man: 210720). MOPD II is characterized by birth weight (14.3 ± 7.7 SD below the population mean and head circumference 8.5 ± 2.1 SD below the population mean, as well as a variety of associated systemic bone and dental anomalies, but there are not any actual cut-off values for diagnosis [18–20]. Our patient’s features differ from those of individuals with MOPD II. However, as our patient has mandibular hypoplasia (Fig. 1a), we speculate that this phenotype was secondarily caused by jaw osteolysis, similar to the phenotypes of MOPD II patients with micrognathia and retrognathia [18–20]. Genetic data from 25 German MOPD II patients, including three families of Turkish origin, were used in the first mutational analysis of *PCNT*, which revealed homozygous and compound heterozygous *PCNT*-null mutations (four splice-site mutations, two small insertions, 10 small deletions, and one exon deletion) [20]. In addition, one Colombian MOPD II patient (nonsense mutation, c.C1468T, in exon 10) and members of one Chinese MOPD II family (small deletions in exons 30 and 41) had apparently homozygous null mutations [21, 22]. *PCNT*-null fibroblast cells derived from MOPD II patients show that a loss of PCNT function induces abnormal mitotic morphology; however, the pathogenic effect of *PCNT* variants in skeletal disorders remains unclear [20].

The PCNT protein has two transcriptional isoforms in humans and mice: the full-length pericentrin protein (isoform 1) and an alternatively spliced form that lacks the N-terminal amino acids (isoform 2), the structure of which has not been determined (Fig. 5b) [23]. However, the molecular mobility of each isoform has not been determined in humans. In vitro and in vivo assays are necessary to identify the functional effect of p.C721R/p.C839R variant.

Bone biopsy is a useful tool for identifying the mechanism of skeletal disorders and determining treatment. Although bisphosphonates and drugs for primary diseases are used in the treatment of systemic bone disease [14, 24, 25], local osteolysis does not require particular therapy; however, patients often need reconstructive surgery [1, 3, 13, 26]. In the patient described herein, we did not perform a biopsy due to the risk of fracture. Thus, this patient was not diagnosed with a novel disorder, and we were unable to perform curative treatment. We consider that dental infection is a serious risk factor for the promotion of osteolysis in this patient and ultimately opted for conservative treatment, with the patient undergoing dental maintenance. Intensive follow up including X ray images if necessary and panoramic X ray is performed once every 3 months. If a mandibular fracture were to appear, reconstructive surgery would be necessary.

The WES results presented in this study suggest that the osteolysis in this patient represents a new disease related to the presence of a variant in *PCNT*. Further investigations are required to determine the function of PCNT and identify the mechanism behind acquired local bone osteolysis.

## Data Availability

The datasets generated and/or analyzed during the current study are available from the corresponding author on reasonable request.

## Abbreviations

MOPD II: Microcephalic osteodysplastic primordial dwarfism type II
WES: Whole exome sequencing
